# Nanoparticle-in-Hydrogel Delivery System for the Sequential Release of Two Drugs

**DOI:** 10.3390/pharmaceutics17010127

**Published:** 2025-01-17

**Authors:** Demian van Straten, Jaime Fernández Bimbo, Wim E. Hennink, Tina Vermonden, Raymond M. Schiffelers

**Affiliations:** 1 CDL Research, University Medical Center Utrecht, 3584CX Utrecht, The Netherlands; j.fernandezbimbo-3@umcutrecht.nl (J.F.B.); r.schiffelers@umcutrecht.nl (R.M.S.); 2 Department of Pharmaceutics, Utrecht Institute for Pharmaceutical Sciences, Utrecht University, 3584CG Utrecht, The Netherlands; w.e.hennink@uu.nl (W.E.H.); t.vermonden@uu.nl (T.V.)

**Keywords:** hydrogel, nanoparticle, liposome, micelle, combination therapy, local delivery

## Abstract

Background/Objectives: Glioblastoma is the most common and lethal primary brain tumor. Patients often suffer from tumor- and treatment induced vasogenic edema, with devastating neurological consequences. Intracranial edema is effectively treated with dexamethasone. However, systemic dexamethasone requires large doses to surpass the blood brain barrier in therapeutic quantities, which is associated with significant side effects. The aim of this study was to investigate a biodegradable, dextran-hydroxyethyl methacrylate (dex-HEMA) based hydrogel, containing polymeric micelles loaded with dexamethasone and liposomes encapsulating dexamethasone phosphate for localized and prolonged delivery. Methods: Poly(ethylene glycol)-*b*-poly(*N*-2-benzoyloxypropyl methacrylamide (mPEG-*b*-p(HPMA-Bz)) micelles were loaded with dexamethasone and characterized. The dexamethasone micelles, together with dexamethasone phosphate liposomes, were dispersed in an aqueous dex-HEMA solution followed by radical polymerization using a photoinitiator in combination with light. The kinetics and mechanisms of drug release from this hydrogel were determined. Results: The diameter of the nanoparticles was larger than the mesh size of the hydrogel, rendering them immobilized in the polymer network. The micelles immediately released free dexamethasone from the hydrogel for two weeks. The dexamethasone phosphate loaded in the liposomes was not released until the gel degraded and intact liposomes were released, starting after 15 days. The different modes of release result in a biphasic and sequential release profile of dexamethasone followed by dexamethasone phosphate liposomes. Conclusions: The results show that this hydrogel system loaded with both dexamethasone polymeric micelles and dexamethasone phosphate loaded liposomes has potential as a local delivery platform for the sequential release of dexamethasone and dexamethasone phosphate, for the intracranial treatment of glioblastoma associated edema.

## 1. Introduction

Glioblastoma is the most common, as well as the most aggressive, type of brain cancer [1]. Despite intensive treatment, patient prognosis is dismal, with a median survival of 14 months after diagnosis. The current standard of care comprises maximal gross surgical tumor resection, followed by a combination of radio- and chemotherapy [1,2]. Nevertheless, tumor recurrence is the rule rather than the exception, and the progression of the disease is often inevitable.

The chemotherapeutic treatment of glioblastoma has barely changed over the last two decades, mostly due to the protective blood–brain barrier which prevents nearly all systemically administered drugs from sufficient disposition in the brain to reach therapeutic concentrations. In response, researchers have investigated the improvement of drug delivery via implantable devices, which have been tested in both preclinical and clinical studies [3,4,5,6]. The opening of the skull for the tumor resection provides a good opportunity to access the brain, and the surgical removal of the tumor conveniently provides space to install such drug delivery devices. This had led to the development of the gliadel wafer, a polyanhydride-based depot that is implanted in the tumor resection cavity and releases the loaded chemotherapeutic carmustine to achieve high local drug concentrations, without systemic exposure to the drug. However, the wafers are rarely able to sufficiently cover the cavity wall and their rigidity does not match with the soft brain tissue, which can lead to mechanically induced damage [7]. Moreover, 95% of the loaded carmustine is released in the first 7 days after implantation, which is not in proportion to the 6–8 weeks of degradation time of the matrix, and, as a result, the empty matrix can stress or even damage brain tissue without providing therapeutic benefit [7,8,9].

As an alternative, hydrogels have been proposed as drug delivery systems for intracranial applications [10]. Hydrogels are three-dimensional polymeric networks capable of absorbing large quantities of water and have mechanical properties that resemble those of natural living tissues. They are, generally speaking, biocompatible and their properties are tailorable particularly by the choice of the building blocks, and their initial water content and crosslink density, making them useful materials for various biomedical and pharmaceutical applications [11]. Particularly, they have shown great promise as drug delivery platforms that allow a local and prolonged release of a variety of therapeutics [12]. These characteristics also make them interesting candidates for the treatment of glioblastoma [13] and several preclinical studies have shown the efficacy of hydrogels loaded with single [14,15] and multiple [16,17] therapeutic compounds via a controlled release. Combining drugs can help overcome the therapy resistance often seen in glioblastoma, thereby improving the treatment outcome [18].

Hydrogels are often based on natural hydrophilic polysaccharides such as hyaluronic acid, alginate, and dextran, as these polymers are easily accessible and provide good possibilities for chemical modification due to the presence of functional groups. Hydroxyethyl methacrylate (HEMA)-modified dextran (dex-HEMA) was used for the development of biocompatible and biodegradable hydrogels, with tunable degradation kinetics and drug release properties [19,20,21]. An aqueous solution of dex-HEMA can be converted into a hydrogel by radical polymerization using a suitable photoinitiator activated by light. This results in easy in situ gelation after the surgical removal of the glioblastoma tumor and the gel, thus, matches very well the available space provided by the resection cavity. The hydrolysis-based degradation of dex-HEMA hydrogels ensures that there is no extra surgery needed to remove the gel once it has released the loaded drugs.

Hydrophilic drugs solubilized in the dex-HEMA gels show release kinetics that vary from hours to months, depending on the properties of both the gel and the loaded drug [19,22,23]. To further modulate the release kinetics, the drugs can be loaded into nanoparticles such as dendrimers, liposomes, and polymeric micelles, which, in turn, can be entrapped in the hydrogel matrix [24]. These nanoparticles provide the possibility of loading both hydrophobic and hydrophilic therapeutics in the hydrogel depot, thereby greatly improving the range of available treatment options for an otherwise difficult to reach tumor such as glioblastoma.

A particularly interesting compound for the treatment of glioblastoma is the corticosteroid dexamethasone, used to reduce edema formation in patients [25,26]. Symptom relief following dexamethasone administration is generally achieved within hours, with maximal improvement within 24–72 h [27]. However, its use is controversial as the systemic and prolonged administration of high doses of dexamethasone, needed to achieve therapeutic concentrations in the brain, results in severe side effects that can even shorten patient survival [28,29,30]. Interestingly, the local delivery of dexamethasone has been shown to reduce the dosing and associated side effects whilst still benefitting from its therapeutic effect in vivo [31]. A hydrogel that locally releases dexamethasone for three days to two weeks would provide an attractive treatment modality for glioblastoma patients suffering from intracranial edema, according to clinical practice guidelines [32].

It was shown that dex-HEMA hydrogels loaded with liposomes yielded a system with drug/liposome release kinetics depending on the hydrogel crosslink density and the thereon dependent gel degradation time [33]. When the size of the liposomes exceeds the mesh size of the polymer network, the liposomes are unable to initially diffuse through the gel and will only be released upon sufficient gel swelling and/or the degradation of the polymer network. When the drug is present in the aqueous liposome core and is unable to permeate through the liposomal bilayer, there is no drug release after the formation of the gel and before extensive gel degradation occurs. However, this release profile is not optimal for the management of cerebral edema, as it is crucial for the drug to be available immediately post-surgery. A hydrogel formulation is therefore needed that releases dexamethasone (either dissolved/dispersed in the hydrogel matrix or loaded in a suitable nanocarrier) directly after implantation into the tumor cavity.

Recently, it was shown that poly(ethylene glycol)-*b*-poly(*N*-2-benzoyloxypropyl methacrylamide (mPEG-*b*-p(HPMA-Bz)) micelles can be loaded with a variety of hydrophobic compounds via π–π stacking and hydrophobic interactions [34,35,36,37]. The micelles provided a sustained release of the encapsulated drugs for varying periods of time depending on the properties of the drug and the micelles [37]. The aim of this study is to develop a dex-HEMA-based hydrogel that releases dexamethasone directly after implantation and that continues to release this drug for two to four weeks as per clinical guidelines [32]. The initial release is achieved by the entrapment of mPEG-*b*-p(HPMA-Bz) micelles loaded with dexamethasone in the hydrogel matrix. Liposomes loaded with the hydrophilic dexamethasone phosphate were co-encapsulated in the micelle-in-hydrogel to ensure the release of the drug-loaded liposomes during the degradation of the hydrogel and ideally shortly after the completion of the release of dexamethasone from the micelles, to ensure long-lasting therapeutic concentrations of this anti-inflammatory drug at the site of action.

## 2. Materials and Methods

### 2.1. Materials

Dulbecco’s phosphate-buffered saline (PBS^1^) (KCl 0.2 g/L, KH_2_PO_4_ 0.2 g/L, NaCl 8.0 g/L, Na_2_HPO_4_ 1.15 g/L), dexamethasone, bovine serum albumin (BSA), lithium phenyl-2,4,6-trimethylbenzoylphosphinate (LAP), cholesterol, uranyl oxalic-acetate, carbonyldiimidazole (CDI), dextran (Mw 40 kDa), NaCl, KCl, NA_2_HPO_4_·H_2_O, KH_2_PO_4_, and methanol were purchased from Sigma-Aldrich, Zwijndrecht, The Netherlands. The mPEG_5kDa_-*b*-p(HPMA-Bz)_17.1kDa_ block copolymer was synthesized as described previously [38]. Tetrahydrofuran (THF) and acetonitrile (MeCN) were obtained from Biosolve, Valkenswaard, The Netherlands. 2-Hydroxyethyl methacrylate was purchased from Fluka, Charlotte, NC, USA. Chloroform was purchased from Merck, Darmstadt, Germany. Dexamethasone phosphate was obtained from Fagron, Capelle aan den Ijssel, The Netherlands. Dipalmitoyl-phosphatidylcholine (DPPC) and poly(ethylene glycol) 2000-distearoyl-phosphatidyl-ethanolamine (PEG(2000)-DSPE) were obtained from Lipoid GmbH, Ludwigshafen am Rhein, Germany. Dex-HEMA with degree of substitution (DS, the number of HEMA groups per 100 glucopyranose groups of dextran) of 5 was prepared as described previously [39].

### 2.2. Preparation of Micelles

**Figure 1:**
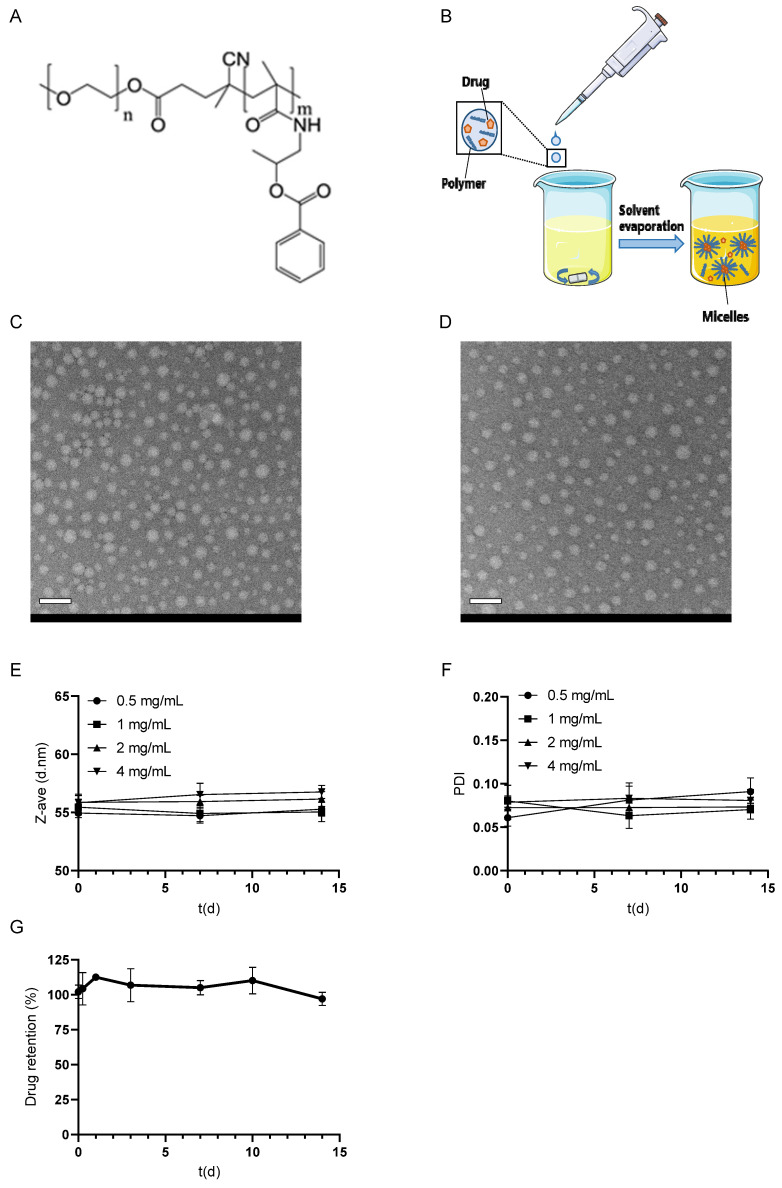
Storage stability of dexamethasone-loaded mPEG-*b*-p(HPMA-Bz) micelles. Chemical structure of mPEG-*b*-p(HPMA-Bz) (from Bresseleers et al., [45]) (**A**). Schematic representation of micelle formation via nanoprecipitation (**B**). TEM images of 1 mg/mL dexamethasone-loaded (**C**) and empty (**D**) micelles. The scale bar represents 100 nm. The size (**E**) and PDI (**F**) of micelles with a LC of 1.5, 3.1, 5.1, and 8.1% dexamethasone (0.5, 1, 2, and 4 mg/mL, respectively) over time during storage at 4 °C in PBS (*n* = 5). Retention of dexamethasone in mPEG-*b*-p(HPMA-Bz) micelles with LC 3.1% in PBS during storage at 4 °C (*n* = 3) (**G**). Data are presented as mean ± SD.

A nanoprecipitation method was used for the preparation of mPEG-*b*-p(HPMA-Bz) micelles as described previously [36,38,40]. In short, the mPEG_5kDa_-*b*-p(HPMA-Bz)_17.1kD_ block copolymer (chemical structure shown in Figure 1A) was dissolved in THF at a concentration of 30 mg/mL. To prepare drug-loaded micelles, dexamethasone was co-dissolved in this polymer solution at feed concentrations between 0.5–4 mg/mL. Next, 1 mL polymer/drug solution was added dropwise to 1 mL milliQ, whilst stirring. Afterwards, THF was removed by evaporation in a fume hood overnight. The volume of the micellar suspension was then adjusted to 0.9 mL with milliQ, followed by the addition of 100 µL 10× PBS (PBS^2^) (1.37 M NaCl, 27 mM KCl, 92 mM NA_2_HPO_4_·H_2_O, 17.6 mM KH_2_PO_4_). Subsequently, the suspension was filtered using 0.45 μm regenerated cellulose membranes (Phenomenex, Torrance, CA, USA) to remove unencapsulated, precipitated drug.

### 2.3. Characterization of Micelles

The mean Z-average particle size and polydispersity index (PDI) of empty and dexamethasone-loaded micelles was determined by dynamic light scattering (DLS). The micellar suspension obtained as described in the section ‘Preparation of micelles’ was diluted 1:19 (*v*/*v*) in PBS^1^ and analyzed at 25 °C using a ZetaSizer Nano ZS 90 (Malvern Panalytical Ltd., Malvern, UK). The dexamethasone content was determined by ultra-high-performance liquid chromatography (UPLC) as described in the section ‘UPLC analysis’. The dexamethasone encapsulation efficiency (EE) and loading capacity (LC) were calculated as follows:

EE = measured amount of dexamethasone/amount of dexamethasone added × 100%(1)

LC = loaded amount of dexamethasone/(amount of polymer + added amount of loaded dexamethasone) × 100%(2)

### 2.4. TEM Analysis

Micelles were immobilized on carbon-coated formvar grids (Copper 100 mesh hexagonal grids, Veco-Stork, Eerbreek, The Netherlands). In short, the grids were placed on top of 20 µL droplets of empty or dexamethasone-loaded micelles (30 mg/mL polymer) and incubated for 10 min at room temperature. The grids were washed three times with PBS^1^ before floating the grids a droplet of 1% (*v*/*v*) glutaraldehyde (Polysciences Inc., Warrington, PA, USA) in PBS^1^ for 5 min. Afterwards, the grids were washed eight times with water to remove phosphate salts and glutaraldehyde. Subsequently, the grids were placed in uranyl oxalic-acetate (pH 7, SPI supplies, West Chester, PA, USA) for 10 min and excess uranyl oxalic-acetate was removed with blotting paper. Next, the grids were placed on a drop of ice-cooled methyl cellulose-uranyl acetate (pH 4, 0.4% uranyl oxalic-acetate) and incubated for 10 min. Finally, the grids were air-dried and imaged on a JEOL3 80 KV electron microscope (JEOL Ltd., Tokyo, Japan).

### 2.5. Storage Stability of Micelles

The colloidal stability of the micelles was determined during storage at 4 °C. In short, freshly prepared micelles (30 mg/mL polymer loaded with 1 mg/mL dexamethasone in PBS^1^) were stored at 4 °C, and, at day 0, 7, and 14, 10 µL samples were taken and diluted with 190 µL PBS^1^. Their size and PDI were measured by DLS as described previously in the section ‘Characterization of micelles’. This process was repeated at 37 °C to investigate whether micelles retained their size during release studies at this temperature, with samples taken at day 0, 3, 7, 10, and 14.

To determine the retention of dexamethasone in the micelles during storage, the micelle suspension (1 mg/mL dexamethasone and 30 mg/mL polymer) was diluted with PBS^1^ 1:4 (*v*/*v*) and incubated at 4 °C. At predetermined time intervals, samples of the micelle suspensions were spun down for 10 min at 5000× *g* to pellet unencapsulated/precipitated dexamethasone and 10 µL samples were taken from the supernatant and added to 90 µL MeCN. Samples were stored at −20 °C until further processing and analysis.

### 2.6. Release of Dexamethasone from the Micelles

To determine the release of dexamethasone from the micelles, micellar suspensions (1 mg/mL dexamethasone and 30 mg/mL polymer in 1× PBS^2^) were mixed 1:3 *v*/*v* with either PBS^1^ or PBA (PBS^1^ with 45 mg/mL BSA) and transferred into a 1 mL Float-a-lyzer device (100 kDa cut-off, VWR International BV, Amsterdam, The Netherlands). The BSA in PBA can bind dexamethasone, thereby improving its solubility. The dialysis device was placed in a closed reservoir containing 40 mL PBS^1^ or PBA and kept at 37 °C. As the solubility of dexamethasone in water is ~90 mg/L at 25 °C [41], the reservoir can dissolve ~3.6 mg dexamethasone. Since the samples in the dialysis device contained 250 µg dexamethasone, sink conditions were met in both PBS^1^ and PBA. At predetermined time points, 10 µL samples were taken from the dialysis device, added to 90 µL MeCN, and stored at −20 °C until further processing as described previously [37].

### 2.7. Preparation of Dexamethasone Phosphate-Loaded Liposomes

Dexamethasone phosphate-loaded liposomes were prepared as described by Deshantri et al. [42]. In short, DPPC, PEG(2000)-DSPE, and cholesterol were dissolved in ethanol in a 62.5:4.8:32.7 molar ratio at a total lipid concentration of 500 mg/mL. The obtained solution of lipids was subsequently injected into an aqueous solution (1:9 *v*/*v*) of dexamethasone phosphate disodium (100 mg/mL). Subsequently, the crude liposome suspension was extruded to achieve the desired liposome size. Ethanol and non-encapsulated dexamethasone phosphate were removed via ultrafiltration and replaced by phosphate-buffered 0.9% saline (pH 7.4). The lipid concentration of the obtained liposomal suspension was measured using high-performance liquid chromatography. The dexamethasone phosphate content in the suspension was determined by UPLC as described in the section ‘UPLC analysis’. Liposome size was determined by dynamic light scattering.

### 2.8. UPLC Analysis

Dexamethasone and dexamethasone phosphate concentrations were quantified using a Waters ACQUITY system (Waters Associates Inc., Milford, MA, USA) equipped with a C18 column (ACQUITY UPLC BEHC18 1.7 μm, 2.1 × 50 mm). A mobile phase existing of 25% MeCN in 75% miliQ and 0.01% phosphoric acid (Merck, Darmstadt, Germany) was used.

Micelle suspensions were diluted 1:9 (*v*/*v*) with MeCN and vortexed to dissolve the micelles and to solubilize the loaded dexamethasone. Samples were subsequently spun down for 10 min at 5000× *g* at room temperature. Sample supernatants were diluted 1:1 with filtered PBS^1^ before measuring dexamethasone content. Samples with known dexamethasone concentrations (3–200 µg/mL) underwent the same procedure for calibration. Samples with dexamethasone concentrations > 200 µg/mL were diluted to fall within the range of the calibration curve. The flow rate was 1 mL/min and the injection volume was 2.5 μL. Absorbance was detected at 254 nm over a 2 min run time.

For samples containing liposomes, dexamethasone phosphate was separated from the lipids using a Bligh and Dyer extraction [43]. In short, 100 µL sample was mixed with 225 µL methanol and 125 µL chloroform, followed by 10 s of vortexing, before another 125 µL PBS^1^ and 125 µL chloroform were added. Subsequently, the mixture was centrifuged for 5 min at 3500× *g* before collecting the sample from the upper aqueous phase. Samples of known dexamethasone phosphate concentrations (3–200 µg/mL) were subjected to the same isolation method for calibration. The flow rate was 1 mL/min and injection volume was 7.5 μL. Absorbance was detected at 254 nm over a 1 min run time.

To determine the dexamethasone and/or dexamethasone phosphate concentration of the dex-HEMA precursor solution in which the micelles and liposomes were dispersed, the solution was first diluted 1:10 (*v*/*v*) in PBS^1^. This obtained solution was then mixed 1:1 (*v*/*v*) with MeCN, vortexed well, and subsequently spun down for 10 min at 5000× *g* at room temperature. The concentration of the drug(s) in the supernatants was subsequently determined using UPLC. For dexamethasone phosphate, the diluted dex-HEMA solution underwent the Bligh and Dyer extraction [43] and the aqueous fraction was analyzed by UPLC.

### 2.9. Hydrogel Release Studies

Dex-HEMA was dissolved at a concentration of 125 mg/mL in either PBS^1^, a suspension of dexamethasone-loaded micelles (30 mg/mL polymer, 1 mg/mL dexamethasone) for micelle-loaded gels, or a 1:1 (*v*/*v*) mixture of dexamethasone-loaded micelle suspension (30 mg/ mL polymer and 1 mg/mL dexamethasone) and dexamethasone phosphate-loaded liposome suspension (10.2 mg/mL total lipids and 0.9 mg/mL dexamethasone phosphate) for a dual-loaded gel. Subsequently, 100 µL LAP (10 mg/mL) was added to 900 µL of the dex-HEMA solution and mixed well. Aliquots of 100 µL were transferred into glass vials (8 × 40 mm) after which dex-HEMA was photopolymerized using a blue light source (50 W, 395 nm miniLED, SJLA LTD., Zhongshan, China) (1 min/gel). Next, 900 µL of PBS^1^ was added on top of the formed gel as release medium. The gels were subsequently incubated at 37 °C. Per time point, 500 µL release medium was collected and replaced with an equal amount of fresh medium. Samples were stored at −20 °C until further analysis. All conditions were tested in triplicate. Before measuring dexamethasone (phosphate) concentrations, the stored samples were thawed and 50 µL was mixed with 100 µL MeCN and vortexed vigorously. Afterwards, 50 µL PBS^1^ was added and the samples were subsequently spun down for 10 min at 5000× *g* at room temperature. Dexamethasone and dexamethasone phosphate concentrations in the supernatants were quantified as described in the section ‘UPLC analysis’. For the gel degradation studies, gels were prepared in glass vials as described above. The glass vials were weighed before and after the gels were prepared, giving the starting weight of the gels. Next, the gels were topped with 900 µL PBS^1^ and incubated at 37 °C. At different time points, the supernatant was removed and the weight of the vials including swollen or partly degraded gel was measured. Degradation/swelling is expressed as Wt/W0 × 100, where Wt = gel weight at time t and W0 is the weight of the gel at t = 0. Degradation of the gels was tested in triplicate.

### 2.10. Rheological Characterization of Hydrogels

The photopolymerization kinetics of dex-HEMA (125 mg/mL) dissolved in PBS^1^ or micelle suspension was investigated via an oscillatory time sweep using a Discovery HDR-2 rheometer (TA-Instruments, Etten-Leur, The Netherlands). The rheometer was connected to a BluePoint 4 UV mercury lamp (λ range 390–500 nm filter, Honle UV technology, Gilching, Germany, 2 W/cm^2^) together with a steel 20 mm Upper Heated Plate (UHP) accessory to maintain a constant temperature of 22 °C. The loading gap was 300 μm and photopolymerization was started 60 s after application of 120 µL sample by turning on the light source which remained on for the duration of the measurement. G′ (storage modulus) and G″ (loss modulus) were recorded at a strain of 0.1% and a frequency of 1 Hz. Gelation time is defined as time from light application until G′ > G″.

### 2.11. Mesh Size

The mesh size of the formed dex-HEMA 10% *w*/*w* hydrogel was calculated using the following equation, Equation (3):

(3) $$\xi ={\left(\frac{G^{\prime }Nav}{RT}\right)}^{-1/3}$$

where ξ is the mesh size in m, G′ is the maximum storage modulus in pascal (Pa), *N_av_* is the Avogadro constant, *R* is the gas constant (8.3144 L·kPa·K^−1^·mol^−1^), and *T* is the absolute temperature in Kelvin [44].

### 2.12. Nanoparticle Tracking Analysis (NTA)

At the predetermined time points, 100 µL samples were collected from the supernatant of the hydrogels during the release studies. Samples were stored at 4 °C until further analysis. The detection and quantification of particles released from the different hydrogel samples were carried out via NTA using a Nanosight NS500 (Malvern Panalytical Ltd., Malvern, UK). Three videos of 30 s at camera level 15 were taken with a detection threshold of 5 and screen gain of 1. Samples were diluted using filtered PBS to achieve 20–100 particles per frame as recommended by the manufacturer.

## 3. Results and Discussion

The aim of this study was to develop a nanoparticle-loaded hydrogel formulation that releases dexamethasone directly after implantation. To this end, a dex-HEMA gel was incorporated with mPEG-*b*-p(HPMA-Bz) micelles, which have demonstrated a capacity for the slow release of various hydrophobic drugs [35,37,40]. The underlying rationale was that the micelles, encapsulated within the hydrogel matrix and loaded with the anti-inflammatory drug dexamethasone, would facilitate the gradual release of the drug into the surrounding tissues.

The micelles were loaded with dexamethasone by dissolving the drug in a THF solution of mPEG-*b*-p(HPMA-Bz) (chemical structure shown in Figure 1A), followed by dropwise adding the dexamethasone/polymer solution into water to form micelles via nanoprecipitation (Figure 1B), followed by solvent evaporation [35].

Table 1 shows that, at a feed concentration of 0.5 and 1 mg/mL, the EE of dexamethasone was >90%, demonstrating the almost quantitative loading of the drug in the micelles. The corresponding LC at a 1 mg/mL feed concentration was 3.1%. The EE decreased with higher feed concentrations of dexamethasone, reaching ~60% at 4 mg/mL of dexamethasone. The corresponding LC at this feed concentration was 8.1%. Table 1 further shows that, independent of the amount of the loaded drug, the diameter of mPEG_5kDa_-*b*-p(HPMA-Bz)_17.1kD_ micelles as determined by DLS was 56 ± 1 nm, similar to the previously described mPEG_5kDa_-*b*-p(HPMA-Bz)_17.1kD_ micelles formed via nanoprecipitation [36,38]. The PDI was ≤0.08 for the different formulations, indicating narrow size distributions. The micelle size distribution by intensity can be found in the Supplementary Data (Figure S1).

The TEM analysis showed that both empty micelles and micelles loaded with dexamethasone were spherical, with a diameter of around 50 nm and a relatively uniform size distribution, confirming the data obtained by the DLS analysis. No obvious structural differences were observed between dexamethasone-loaded (Figure 1C) and empty micelles (Figure 1D) in accordance with previous studies of mPEG-*b*-p(HPMA-Bz) micelles loaded with other drugs [34,45].

Independent of dexamethasone loading, the micelles retained their size (Figure 1E) and PDI (Figure 1F) during storage in PBS at 4 °C for at least 14 days. Importantly, no dexamethasone leakage was seen from the micelles for two weeks during storage at 4 °C (Figure 1G). These observations demonstrate that the dexamethasone-loaded micelles can be prepared in advance and stored at 4 °C until use.

**Figure 2:**
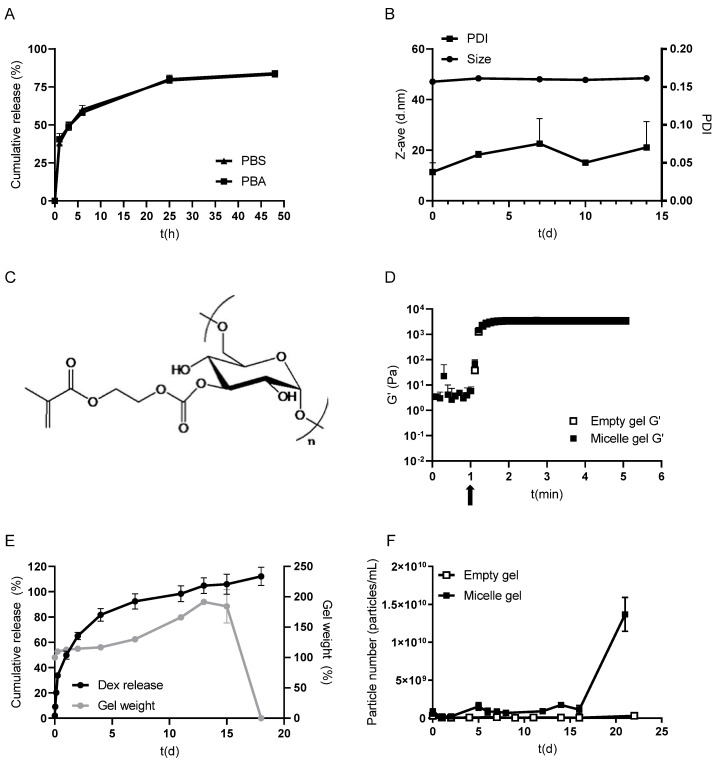
Characteristics of dexamethasone-loaded mPEG-*b*-p(HPMA-Bz) micelles (LC 3.1%) and micelle-loaded dex-HEMA (10 wt% and DS 5) hydrogels. Release of dexamethasone from micelles in PBS and PBS supplemented with 45 mg/mL BSA (PBA) over 48 h at 37 °C and under sink conditions (**A**). The size and PDI of dexamethasone-loaded micelles incubated for 14 days in PBS at 37 °C (**B**). Chemical structure of dex-HEMA (from Pescosolido et al. [23]) (**C**). Storage modulus G′ of empty and micelle-loaded dex-HEMA hydrogels as a function of time. Blue light irradiation was initiated 1 min after the sample was introduced in the rheometer, as indicated by the arrow (**D**). The cumulative release of dexamethasone (left Y-axis) and gel weight (right Y-axis) from micelle-loaded dex-HEMA hydrogels as a function of time (**E**). The number of particles per mL measured in the supernatant of empty and dexamethasone-micelle-loaded dex-HEMA hydrogels over time (**F**). Data are presented as mean ± SD (*n* = 2–3).

For further experiments, micelles loaded with 3.1% dexamethasone were used, since it was shown in a previous study that the overloading of the micelles reduces drug retention over time [40]. The micelles released around 85% of their content within 48 h in PBS at 37 °C (Figure 2A). The release was also determined in PBS supplemented with BSA (PBA) to provide a solubilizer since albumin can bind dexamethasone [46,47]. However, the addition of albumin did not change the release kinetics of dexamethasone (Figure 2A). In a previous study, the faster release of certain drugs in the presence of albumin was observed [37]. In the present study, BSA did not affect the release kinetics of dexamethasone from the micelles, likely because, for both media, the sink conditions were amply met. To explain this, the solubility of dexamethasone is ~90 µg/mL in water at 25 °C [44]. Because the 40 mL release buffer used here is over 10× the volume needed to fully dissolve the 250 µg of dexamethasone loaded in the micelles, the sink conditions are ensured.

Similar to the stability of micelles at 4 °C, the micelles had good colloidal stability at 37 °C as the size and PDI did not change over 14 days (Figure 2B). The relatively low loading capacity (Table 1) and fast release of dexamethasone from the micelles (Figure 2A) can be explained by the lack of aromatic groups in dexamethasone and its relatively low log P (~1.92, [48]), two factors that were previously shown to be important for micelle loading and drug retention in the micelle core [37]. It is noted, however, that the resulting release properties are favorable for the intended local delivery approach as the immediate release of dexamethasone upon hydrogel implantation is desired.

The micelles were loaded into dex-HEMA hydrogels, formed by photocrosslinking aqueous dex-HEMA (chemical structure depicted in Figure 2C) solutions using LAP as the photoinitiator. This photoinitiator in combination with blue light irradiation does not cause the photodegradation of dexamethasone, as opposed to the UV light needed for a photoinitiator such as Irgacure 2959 (Figure S2). A rheological analysis showed that dex-HEMA dissolved in an aqueous solution, either in the absence or presence of micelles, had a gelation time with G′ > G″ within 0.1 min after illumination (Figure 2D), which is in line with previous findings [23]. The addition of micelles did not significantly affect the time it took to reach the plateau value of G’ for the gels (empty at 1.04 min vs micelle-loaded at 1.08 min). The storage modulus was, within the experimental error, the same for both gels (empty gel at 3.5 kPa vs gel loaded with micelles at 3.6 kPa; Figure 2D). Based on the G’, the calculated mesh size ξ of the gels (Equation (3)) was 10.6 nm, demonstrating that the micelles (diameter of 55 nm; Table 1) were immobilized in the hydrogel matrix and can, therefore, only be release from the gel after the substantial degradation of the polymer network.

Micelles dispersed in the dex-HEMA hydrogel released 65% of the loaded dexamethasone during the first two days, and the remaining drug was released over the next 18 days (Figure 2E). This release pattern slightly differs from Figure 2A, in which it is shown that micelles dispersed in PBS released 85% of their content during the first two days. The first 24–72 h are crucial for treating the surgery-induced edema with dexamethasone [27] and it is recommended to discontinue or taper dexamethasone administration after three days [32,49]. The release profile of dexamethasone from the hydrogel loaded with polymeric micelles in which the drug is solubilized thus aligns very well with the guidelines of corticosteroid use in glioblastoma treatment.

Particle measurements in the gel supernatant were carried out over time to investigate whether intact micelles with dexamethasone solubilized in their core were released from the gel, or whether dexamethasone was released from the micelles dispersed in the hydrogel and subsequently diffused from the gel into the surrounding medium. During the first ~15 days, a very low number of particles was detected in the supernatant of both the micelle-loaded gels and the empty gels. When the gels start to disintegrate after 15 days, a sudden significant increase in particle concentration was observed for micelle-loaded gels compared to empty gels (Figure 2F), demonstrating the release of the originally entrapped micelles from the hydrogel. The size of the particles after their release from the hydrogel was 49 ± 5 nm as determined by NTA, which is in line with the size of the micelles dispersed in PBS (Table 1). The degradation characteristics of the dex-HEMA hydrogel (with a DS 5 and initial water content of 90%) observed in the present study is in line with a previous study [19].

Figure 2E confirms that micelles retained their integrity in the hydrogel and released the loaded dexamethasone before they were released from the hydrogel, as a nearly quantitative release of dexamethasone was observed before the degradation of the gel and release of the micelles.

**Figure 3:**
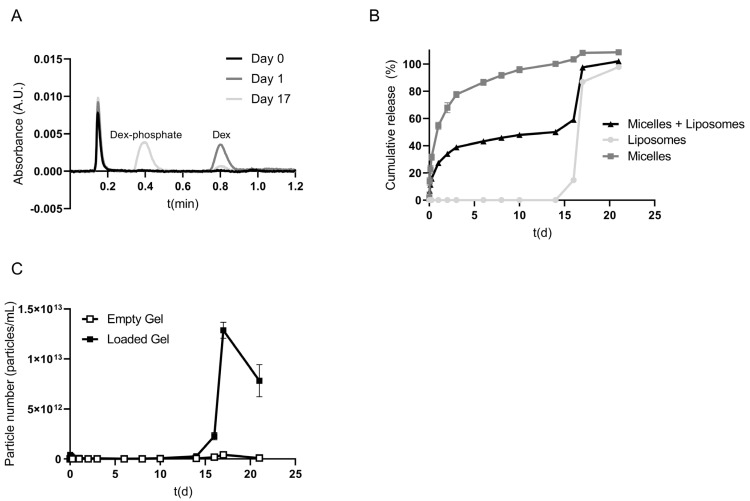
Release of dexamethasone, dexamethasone phosphate, and nanoparticles from micelle- and liposome-loaded dex-HEMA (10 wt% and DS 5) hydrogels. Representative UPLC chromatograms depicting the detection of dexamethasone phosphate (retention time ~0.4 min) and dexamethasone (retention time ~0.8 min) in hydrogel release samples at t = 0, day 1, and day 17. The peak before 0.2 min represents the injection peak (**A**). Cumulative release of dexamethasone (grey), dexamethasone phosphate (light grey), and both (black) from a micelle- and liposome-loaded hydrogel formulation (**B**). Number of particles per mL supernatant of empty and nanoparticle-loaded gels over time (**C**). Data are presented as mean ± SD (*n* = 3).

Taken together, the release of dexamethasone from the micelle-loaded hydrogel is dependent on the release of the drug from the micelles, rather than the degradation of the hydrogel and subsequent release of a hydrophilic model drug (calcein) present in the aqueous core of liposomes that are, in turn, entrapped in a dex-HEMA hydrogel [33]. Dexamethasone phosphate-loaded liposomes (total lipid content of 10.2 mg/mL, encapsulating 0.9 mg/mL dexamethasone phosphate) and dexamethasone-loaded micelles (LC 3.1%) were co-encapsulated in the dex-HEMA hydrogel to investigate the feasibility of a sequential drug release. With the described UPLC method, both dexamethasone and dexamethasone phosphate were simultaneously determined, which makes it possible to distinguish between the drug released from both the micelles and the liposomes within the same sample (Figure 3A). The particle concentration in the release samples was also determined.

Figure 3B shows that, similar to the hydrogels loaded with only micelles, ~65% of dexamethasone was released within two days, followed by a slow release of the remaining drug in the following two weeks (Figure 2E). Figure 3B also shows that no dexamethasone phosphate was released until day 15. Starting from day 15, however, a rapid release of dexamethasone phosphate was observed, indicating the release of intact liposomes loaded with dexamethasone phosphate. Figure 3C shows, in accordance with Figure 2F, that when gel degradation starts from day 15, the nanoparticle concentration in the supernatant of the hydrogel sharply increased. The drop in the particle number seen between day 17 and 21 is due to dilution by replacing the removed sample volume with fresh buffer after all the particles were released. The mode size of the particles in the supernatant after release (average of last three timepoints) was 63.5 ± 2.6 nm, which is in between the size of the micelles (55 nm) and the liposomes (120 nm). The results of Figure 3 demonstrate that, by co-encapsulating the micelles and liposomes in the same dex-HEMA hydrogel, it is possible to release the two loaded drugs sequentially. The combination of both nanoparticles within the same gel resulted in the sequential release of, first, dexamethasone-, followed by dexamethasone phosphate-loaded liposomes. After release, the liposomes are available for uptake and endosomal processing by the surrounding cells. Within the endosomes, the lipid bilayer of the liposomes is destabilized, leading to the release of dexamethasone phosphate. The prodrug is subsequently enzymatically converted to dexamethasone by phosphatases.

This study focuses on dexamethasone as an active pharmaceutical ingredient. However, both the micelles and the liposomes were previously loaded by a variety of other therapeutics [34,35,36,37,42,50]. Consequently, many therapeutics are eligible to be combined within this gel to make optimal use of this platform. It is a favorable property to be able to deliver multiple therapeutics using this platform, as clinical results show that a single-drug approach rarely succeeds in treating glioblastoma effectively [18]. In previous studies, it was shown that the release of the micellar- and liposomal-encapsulated drugs can be modulated independently by exploiting the properties of both the hydrogel and the loaded nanoparticles. To explain this, by changing the crosslink density and/or polymer concentration of the gel, the rate at which the gel degrades can be tailored, and, thus, the release of liposomal drug can be modulated [19,33]. Further, the release of the micellar-loaded drug is not dependent on gel degradation, and, therefore, the release characteristics can be modulated by both the type of drug [37] and the composition of the mPEG-*b*-p(HPMA-Bz) block copolymer [40].

## 4. Conclusions

This study describes the development of a biodegradable dex-HEMA hydrogel in which both polymeric micelles and liposomes were entrapped. The micelles were loaded with the hydrophobic drug dexamethasone, whereas the liposomes were loaded with the hydrophilic dexamethasone phosphate. This hydrogel released free dexamethasone and liposomal dexamethasone phosphate in a sequential manner with different release mechanisms. This provides a platform for the localized delivery of multiple therapeutic agents, thereby increasing local drug concentrations and thus reducing the off-target effects. Options to modulate the release kinetics of the micellar-loaded drug and the liposomal-loaded drug are available by tailoring the properties of the hydrogel, particularly the initial water content and crosslink density, and those of the micelles, particularly the type of drug loaded and the molecular weight and composition of the block copolymer.

## Figures and Tables

**Table 1:** Size, PDI, encapsulation efficiency (EE), and loading capacity (LC) of dexamethasone loaded micelles at 30 mg/mL polymer of 3 independently prepared micelle batches.

Dexamethasone Concentration (mg/mL)	Size (nm) (*n* = 2)	PDI (*n* = 2)	EE (%) (*n* = 3)	LC (%) (*n* = 3)
0.0	55 ± 1	0.07 ± 0.01	-	-
0.5	55 ± 1	0.06 ± 0.06	93 ± 6	1.5 ± 0.1
1.0	56 ± 0	0.08 ± 0.07	93 ± 7	3.1 ± 0.2
2.0	56 ± 1	0.07 ± 0.01	77 ± 2	5.1 ± 0.1
4.0	56 ± 1	0.08 ± 0.01	61 ± 6	8.1 ± 0.8

## Data Availability

The data will be made available upon request.

## Supplementary Materials

The following supporting information can be downloaded at: https://www.mdpi.com/article/10.3390/pharmaceutics17010127/s1, Figure S1. The diameter size distribution (in nm) by intensity of micelles loaded with 0, 0.5, 1, 2, or 4 mg/mL dexamethasone, as measured by DLS; Figure S2. Degradation of dexamethasone phosphate (DexP) and dexamethasone (Dex) occurs after exposure to UV-light (A), but not after exposure to blue light (B). Samples of 100 µg/mL dexamethasone phosphate or dexamethasone in PBS^1^ were exposed to the respective light sources for 0, 20, or 60 s and subsequently analyzed by UPLC.
