# Supplementary material for: Nanoparticle-in-Hydrogel Delivery System for the Sequential Release of Two Drugs
Source: Pharmaceutics. 2025 Jan 17;17(1):127. doi: 10.3390/pharmaceutics17010127 (PMC11768762; doi:10.3390/pharmaceutics17010127)
Supplement: Supplementary file 1 [file pharmaceutics-17-00127-s001.zip › pharmaceutics-3416708-supplementary.pdf]

pharmaceutics

Supplementary

Nanoparticle-in-Hydrogel Delivery System for the Sequential Release of Two Drugs

Demian van Straten, Jaime Fernández Bimbo, Wim E. Hennink, Tina Vermonden and Raymond M. Schiffelers

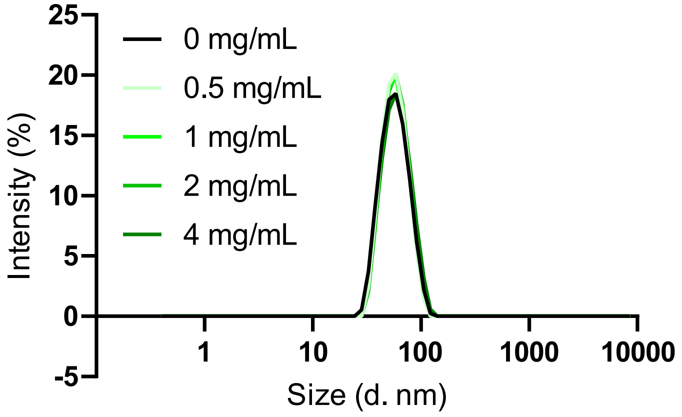

Figure S1. The diameter size distribution (in nm) by intensity of micelles loaded with 0, 0.5, 1, 2 or 4 mg/mL dexamethasone, as measured by DLS.

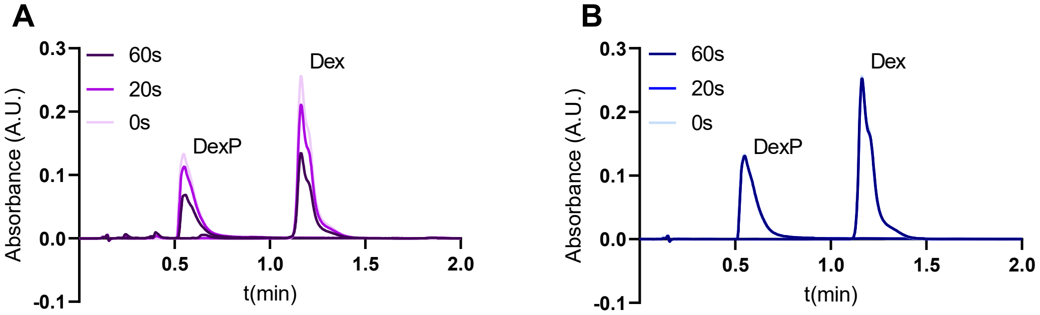

Figure S2. Degradation of dexamethasone phosphate (DexP) and dexamethasone (Dex) occurs after exposure to UV-light (A), but not after exposure to blue light (B). Samples of 100 µg/mL dexamethasone phosphate or dexamethasone in PBS1 were exposed to the respective light sources for 0, 20 or 60 seconds and subsequently analyzed by UPLC.
